# Barriers and Facilitators to Promoting Oral Health Literacy and Patient Communication among Dental Providers in California

**DOI:** 10.3390/ijerph18010216

**Published:** 2020-12-30

**Authors:** Winston Tseng, Elizabeth Pleasants, Susan L. Ivey, Karen Sokal-Gutierrez, Jayanth Kumar, Kristin S. Hoeft, Alice M. Horowitz, Francisco Ramos-Gomez, Miku Sodhi, Jessica Liu, Linda Neuhauser

**Affiliations:** 1 Health Research for Action, School of Public Health, University of California, Berkeley, CA 94720, USA; b_pleasants@berkeley.edu (E.P.); sivey@berkeley.edu (S.L.I.); ksokalg@berkeley.edu (K.S.-G.); jessica.liu@berkeley.edu (J.L.); lindan@berkeley.edu (L.N.); 2 Office of Oral Health, California Department of Public Health, Sacramento, CA 95899, USA; Jayanth.Kumar@cdph.ca.gov; 3 Department of Preventive & Restorative Dental Sciences, School of Dentistry, University of California, San Francisco, CA 94143, USA; Kristin.Hoeft@ucsf.edu; 4 Department of Behavioral Health & Community Health, School of Public Health, University of Maryland, College Park, MD 20742, USA; ahorowit@umd.edu; 5 Division of Pediatric Dentistry, School of Dentistry, University of California, Los Angeles, CA 90095, USA; frg@dentistry.ucla.edu; 6 Shasta Cascade Health Centers, McCloud, CA 96057, USA; miku_sodhi@hotmail.com

**Keywords:** oral health, health literacy, oral health literacy, provider-patient communication, dental providers

## Abstract

Studies demonstrate that dental providers value effective provider-patient communication but use few recommended communication techniques. This study explored perspectives of California dental providers and oral health literacy experts in the United States on use of communication techniques. We conducted a qualitative key informant interview study with 50 participants between November 2019 and March 2020, including 44 dental providers (dentists, hygienists, and assistants) in public or private practice in California and 6 oral health literacy (OHL) experts. We undertook thematic analysis of interview transcripts and descriptive statistics about interviewees from pre-surveys. Dental providers reported frequently speaking slowly, and using simple language and models/radiographs to communicate with patients, while infrequently using interpretation/translation, illustrations, teach-back, or motivational interviewing. Providers reported using only 6 of the 18 American Medical Association’s (AMA) recommended communication techniques and only 3 of the 7 AMA’s basic communication techniques. A majority of providers indicated using one of five oral health assessment and educational strategies. Key barriers to effective communication included limited time, financial incentives promoting treatment over prevention, lack of OHL training, limited plain-language patient education materials, and patients with low OHL knowledge. Dental organizations should prioritize supporting dental providers in effective patient communication practices. Standardizing OHL continuing education, creating an evidence-based OHL toolkit for dental teams, ensuring accessible interpretation/translation services, and incentivizing dental providers to deliver education could improve oral health literacy and outcomes.

## 1. Introduction

In the United States (US) and globally, poor oral health is a major, but preventable public health problem [1,2,3]. Disparities and inequities in oral diseases are also a serious social justice concern, as low-income and marginalized groups are disproportionately impacted by poor oral health [2,3,4,5,6,7,8]. In addition to a lack of access to care, oral health literacy (OHL) is a major factor in these disparities. Research over the past two decades has identified low OHL as a key contributor to poor oral health [9,10,11,12]. The US Department of Health and Human Services in its report Healthy People 2010, defined OHL as “the degree to which individuals have the capacity to obtain, process and understand basic oral health information and services needed to make appropriate health decisions” [13,14]. The concept of OHL has recently been expanded to include the abilities of oral health providers to communicate effectively with patients/caregivers and create “patient-friendly” environments [12]. Improving OHL is a critical challenge for advancing global oral health. The US Healthy People 2030 [15] provides the following new, combined definition of health literacy:

- **Personal health literacy** is the degree to which individuals have the ability to find, understand, and use information and services to inform health-related decisions and actions for themselves and others.
- **Organizational health literacy** is the degree to which organizations equitably enable individuals to find, understand, and use information and services to inform health-related decisions and actions for themselves and others.

Studies of general health literacy (mostly from medical settings) have identified three main health literacy intervention areas and documented overall positive results from: (1) designing easy-to-use communication resources matching people’s literacy and linguistic skills [16,17]; (2) training health providers to use effective communication techniques [16,17]; and (3) developing “patient-friendly” and “shame-free” healthcare environments [18,19,20].

Building upon this foundational health literacy work in medicine, training dental team providers on oral health literate communication techniques is now considered a priority area for research and for development of effective strategies.

Research has examined OHL and communication practices from the perspectives of healthcare providers, including dentists, dental hygienists, nurse practitioners, primary care providers, and pediatricians. Rozier et al. conducted a national survey of 1994 US dentists assessing use of 18 patient communication techniques recommended by the American Medical Association (AMA) [21,22]. The study found that dentists reported using a mean of 7 out of 18 recommended communication techniques, and only 3 out of 7 communication techniques that were considered “basic techniques.” The main techniques reported by two-thirds of respondents were to: speak slowly, use plain language, demonstrate with models/radiographs, and provide caregivers printed handouts to take home. Less than 25% of dentists reported using “teach-back.” This technique is one in which a provider asks a patient to describe—in their own words—a treatment they receive during a clinical visit or what they will do for their treatment or preventive care at home to follow the provider’s guidance [23,24]. Dentists who were older, female, Black/African American, educated outside of the US, and specialists (with the exception of pediatric dentists) were likely to report using more communication techniques. Overall, 73.3% of participating dentists had never taken a health communications course, and 68.5% indicated they would be interested in continuing education (CE) to improve their communication with patients [21].

Besides the single nationwide study conducted by Rozier et al., there have been some state-level studies. Horowitz et al., Koo et al., Weatherspoon et al., and Maybury et al. surveyed oral health providers in Maryland to assess their use of recommended communication techniques [25,26,27,28]. Dentists (general and pediatric), dental hygienists, nurse practitioners, and physicians (family medicine and pediatrics) all reported using an average of 47% or less of the recommended communication techniques. Maybury et al. surveyed general practice and pediatric dentists in private practice to assess the use of 18 AMA-recommended communication techniques, including 7 basic communication techniques. The seven basic communication techniques included five that cover interpersonal communication and two that highlight teach-back. The interpersonal communication items included: Cover two–three concepts at a time; Ask the patient if they would like family/friends in discussions; Draw pictures; Speak slowly; and Use simple language. The teach-back method items included: Ask the patient to repeat back; and Ask the patient to tell you what they will do at home. In Marbury and colleagues’ study, of 1393 general dentists and 169 pediatric dentists who were sent the survey, 38.4% provided responses. About 60% of responding providers reported having taken a communication course. General dentists reported using 7.9 out of the 18 communication techniques and 3.6 out of the 7 basic techniques; pediatric dentists used more techniques (8.4/18 total and 3.8/7 basic techniques). General dentists who reported having taken a communication course had higher use of the 18 communication techniques but not of the 7 basic techniques, while pediatric dentists who reported having taken a communication course had higher reported use both of the 18 communication techniques and the 7 basic techniques. Less than 20% of participants reported using teach-back [28]. Horowitz et al. surveyed a sample of Maryland dental hygienists to assess the use of the same communication techniques. Of the 1259 surveys sent, they received 540 valid responses (42.9% response rate). Dental hygienists reported using an average of 7 out of the 18 communication techniques routinely, and 3.7 out of the 7 basic techniques. Less than 5% reported using all 18 communication techniques. Only one out of three routinely reported using teach-back [25].

Some US states have established oral health initiatives that recommend dental provider OHL training [12,29]. California is the state with the largest number of dental providers in the US: 30,772 dentists [30], 22,800 dental hygienists [31], and 56,840 dental assistants [32]. California is the largest and most diverse state in the US—with 40 million residents (1/8 of the US population). California has a majority non-White population; 27% are immigrants, primarily from Latin America and Asia [33]. This creates a unique context for oral health care and poses OHL challenges for providers and patients. In collaboration with the California Department of Health Care Services, the California Department of Public Health (CDPH) Office of Oral Health (OOH) developed the California Oral Health Plan for 2018–2028, addressing California’s current major issues in oral health and providing guidance to improve oral health and achieve oral health equity [29,34]. One key strategy in this plan is to “provide training and resources to improve dental teams’ communication with patients,” emphasizing steps to assess and build on current resources and skills for providing OHL-based care.

Our research team carried out a study as a part of efforts by the CDPH with the following objectives: (1) to add to the limited literature about dental provider use of recommended OHL communication techniques; (2) to conduct a qualitative California study of dental team providers’ OHL knowledge, perspectives and practices, interests in training, and preferences for training delivery; and (3) to apply findings to inform the development of statewide OHL trainings and resources for dental providers. This study is the first to examine Californian dental providers’ and national OHL experts’ perspectives on provider-patient OHL communication. This study was categorized as exempt from full Institutional Review Board (IRB) review by the IRB at California’s Office of Statewide Health Planning and Development (OSHPD).

## 2. Methods

We conducted a qualitative study, including 50 key informant (KI) interviews and using pre-surveys to collect demographics and use of communication techniques with a sample of dental providers and OHL experts from November 2019 to March 2020. Our KIs included dentists (general dentists and pediatric dentists), dental hygienists, and dental assistants across California (*n* = 44), and national OHL subject matter experts (*n* = 6). Participants were recruited using both purposive and snowball sampling strategies in collaboration with the CDPH OOH, California Dental Association, Dental Board of California, California Dental Hygienists Association, California Dental Assistants Association, as well as other key state and national organizations. Dental providers (general dentists, pediatric dentists, dental hygienists, and dental assistants) were eligible if they currently provided dental care at a federally qualified health center (FQHC)/community clinic or private practice in California. Dental providers were purposively sampled from geographic locations across California including urban, peri-urban, and rural areas. Experts were identified for the study based on their expertise in identifying and shaping state- and national-level OHL-related research, interventions, and/or policies. Potential participants were approached via phone, email, or email introductions from our state and national partners. Dental provider recruitment ceased when data saturation was observed in the concurrent data analyses.

We designed two sets of study instruments: one for dental providers and one for OHL experts. Each set included telephone interview guides and web-based pre-surveys for demographics and communication techniques used. Instruments were pilot-tested with providers and OHL experts. The interview guides and descriptive pre-surveys were developed based on a scoping review of relevant literature and an environmental scan of existing oral health educational materials and programs, as well as an iterative content review. We also examined five oral health assessment and educational strategies in the telephone interview that we identified from our scoping review and environmental scan. Survey questions about communication techniques were adapted from prior OHL surveys assessing provider use of effective communication techniques, primarily the AMA’s 18 communication techniques [22] and 7 basic communication techniques [21,25,26,27,28]. 

In total, 40 out of 44 providers completed the pre-survey independently before their telephone interview. All 44 providers completed the telephone interview. Electronic informed consent was given to the interviewees via email prior to proceeding with the web-based pre-survey and scheduling an interview. The consent information was also repeated verbally, asking them again if they agreed to be interviewed, just before the beginning of the telephone interview. Questions in instruments required yes/no, multiple-choice, or open-ended responses, depending on which portion of data collection is described. The provider pre-survey contained 21 questions and included items related to professional background, work environment, role in patient education, use of types of patient communication techniques including AMA’s 18 communication techniques and 7 basic communication techniques, prior patient communication training, future training preferences, and demographics. The OHL expert pre-survey contained nine questions and included items related to professional background, health literacy and/or OHL experience, and demographics.

Following completion of their web-based pre-survey, providers and OHL experts participated in a 30- to 60-min semi-structured open-ended telephone interview conducted by a member of the research team. The telephone interview allowed participants to elaborate on their opinions and experiences with patient communication and education strategies in general and with disadvantaged patient groups. Provider interviews included 11 questions and assessed items such as experiences with patient education and communication, knowledge of assessment and educational strategies and oral health literacy, gaps in professional training and education, and recommendations on improving patient-provider communication. Communication and educational methods assessed by telephone interviews included the “Tell-Show-Do” approach described and demonstrated as effective by Avenetti et al. [35], the caries risk assessment (CAMBRA), anticipatory guidance, family oral health education (FOHE), motivational interviewing (MI), and teach-back described and promoted by Ramos-Gomez and Ng [36]. Anticipatory guidance, CAMBRA (an assessment tool), and FOHE were included in this exploration to understand how explaining caries risk and anticipatory guidance can be used as educational approaches to improve provider-patient communication especially for parents and caregivers of pediatric populations. Expert interviews included 15 questions and assessed items related to OHL best practices among dental providers, factors that promote or hinder improvements in OHL, gaps in training and professional education, development of an OHL toolkit and materials to use in dental practice, meeting the needs of diverse populations, and innovations and promising practices. Most of the interviews were recorded. Interview notes were taken during all interviews.

Interview transcripts and notes were compiled and organized by KI type for qualitative analysis. Descriptive statistics were used to characterize the survey participants using the pre-survey items. The constant comparative method was used as a technique for the qualitative thematic analysis. This method develops codes, examines relationships and interactions across descriptive and thematic codes, and compares the major themes that emerged from the coding categories [37]. Qualitative analysis probed for parallel themes, particularly looking for provider knowledge and use of oral health assessment and educational strategies and communication barriers and facilitators. The final codebook consisted of descriptive and thematic codes common across the 50 KI interviews. Four researchers independently coded transcripts from the KI interviews. Inter-rater agreement for the first two provider interview transcripts and the first two expert interview transcripts was determined to ensure consistency in coding. If 80% agreement in coding consistency was not reached, the researchers discussed potential issues that arose and reached consensus about these coding issues until consistency was reached. Next, these initial transcripts were recoded and the coding consistency percentage was recalculated. The remaining transcripts were then coded using the updated codebook. The codebook was revised as subsequent transcripts were coded and new codes emerged. Coding consistency was recalibrated as part of this iterative coding process. Code categories were connected and grouped through thematic coding, and the researchers identified major themes from the codes. 

In addition, pre-survey data collected using Qualtrics were imported into Stata (Version 15, StataCorp, College Station, Texas, USA) for descriptive analysis of background information and use of communication techniques on providers. 

## 3. Results

### 3.1. Summary of KI Characteristics

In total, 44 qualitative dental provider key informant interviews and 40 associated pre-surveys were completed. The characteristics of 40 out of the 44 California dental providers (RR = 91%) from the pre-surveys are presented in Table 1. Of the 40 dental providers, 48% of the providers were dentists, with almost equal representation from private practice (25%) and public practice (23%); 30% were dental hygienists; and 23% were dental assistants, with 65% of dental hygienists and dental assistants from private practice settings. Most providers were female (70%). Most identified their race/ethnicity as White (60%) followed by Asian/Pacific Islander (25%), Hispanic/Latino (10%), Black/African American (3%), or “Other.” Providers’ average length of practice was 22 years, with a range from 1.5 to 49 years. All OHL experts (*n* = 6) had backgrounds in both public health and OHL, and two experts were dentists. Most experts were female (67%). All experts were White, and the majority were affiliated with an academic institution (83%), while one worked with a dental association. Experts had between 14 and 48 years working in their field, with an average of 40 years of experience; the majority provided direct service to the community (83%).

### 3.2. Provider Knowledge and Use of OHL Educational and Communication Techniques

In the open-ended qualitative telephone interviews (*n* = 44), providers discussed valuing effective communication and feeling “extremely” or “very” confident in communicating with patients. One dental hygienist illustrated this, saying:

“In hygiene school, it’s one of the many things we do, oral health education (…) I increase my own oral health literacy as new (OHL) studies come out on my own. As a public health professional, oral health is important to me. Even looking at (…) how it affects populations, to me it’s health equity. It’s imperative for all of us to understand this.”

(Dental Hygienist, Public Practice)

Most dental providers interviewed reported that they were familiar with the term “oral health literacy” (OHL) and believed that it was vital to patient communication. While most providers understood that OHL addresses a patient’s ability to understand and act on oral health information, few providers knew that OHL also involves the provider’s ability to communicate with a patient, and the patient-friendliness of the dental environment. The OHL experts affirmed this disparity, commenting that dental providers generally considered themselves good communicators, but they may not tailor their communication style for patient needs or ensure patient understanding and ability to practice positive oral health behaviors at home.

This was echoed in the web-based survey (*n* = 40), where 86% of dental providers who answered the survey items rated their training and competency in patient education and communication as “good” or “very good.” Two-thirds reported having taken a communication course, and 70% expressed interest in receiving continuing education on communication. Half of the providers reported having assessed the “patient-friendliness” of their practice but provided no information about their process to do so.

In terms of educational and communication strategies, most dental providers from the telephone interviews, particularly dental hygienists and dental assistants, considered visual communication and the “Tell-Show-Do” approach to be highly effective for patient/caregiver education. Many indicated that they used a variety of visual communication approaches, including dental models, intraoral cameras, mirrors, pictures, charts, brochures with visuals, and videos. Most dentists reported that dental hygienists and dental assistants provided the majority of patient oral health education. The public practice providers generally described patient education delivered by one designated provider, while the private practices generally described reinforcement of patient education messages by all team members.

The telephone interviews probed dental providers’ awareness and use of five types of oral health assessment and educational strategies: caries risk assessment (CAMBRA) [36], anticipatory guidance, family oral health education (FOHE), motivational interviewing (MI), and teach-back. Many providers lacked understanding of these five types of oral health assessment and educational strategies, particularly private practice dentists, dental hygienists, and dental assistants (Table 2).

Most dental providers interviewed were unaware of or did not use family oral health education, motivational interviewing (MI), or teach-back. Those who knew about, but did not use these approaches, cited them as not being a required standard of practice and/or having limited time for patient education/communication. One dentist described the challenges in using MI in their public practice:

“You talk to the patient and educate them and trying to understand why they need to do certain things (…) Try to use the outcomes to motivate them, to change behavior. (…) Motivational interview takes a lot of time. Not everyone can do it. We have a lot of patients. Need to carve out 5–10 min (for patient communication) is all we can do.”

(Dentist, Public Practice)

We asked all providers about their use of CAMBRA and FOHE despite being primarily intended for use in pediatric populations. Many providers did not know about or use FOHE, while more providers were familiar with and used CAMBRA; providers serving children more consistently reported using these techniques in their practice.

**Figure 1:**
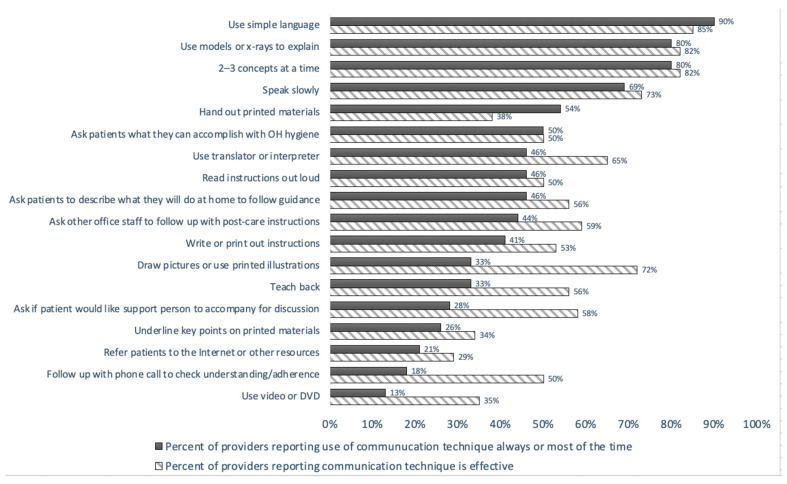
Use and effectiveness of American Medical Association’s 18 communication techniques in practice (*n* = 40). Assessment of provider use and perceived effectiveness of AMA’s 18 communication techniques; see Rozier et al., 2011, for previous assessment of these techniques in practice by oral health providers.

Web-based survey responses from 40 of 44 dental providers addressed their use of and perceived effectiveness of the AMA’s 18 patient communication techniques (Figure 1). A majority of providers indicated they used 6 out of 18 AMA-recommended communication techniques always or most of the time, including simple language (90%), visual demonstration with models or radiographs (80%), presenting two–three concepts at a time (80%), speaking slowly (69%), handing out printed materials (54%), and asking patients what they can accomplish with oral health hygiene (50%). However, most also reported not using some of these communication techniques that they believed were effective, such as drawing pictures or using printed illustrations, using teach-back, using a translator/interpreter, asking other office staff to follow up with post-care instructions, and asking patients if they would like their support person to accompany them to their appointment.

**Figure 2:**
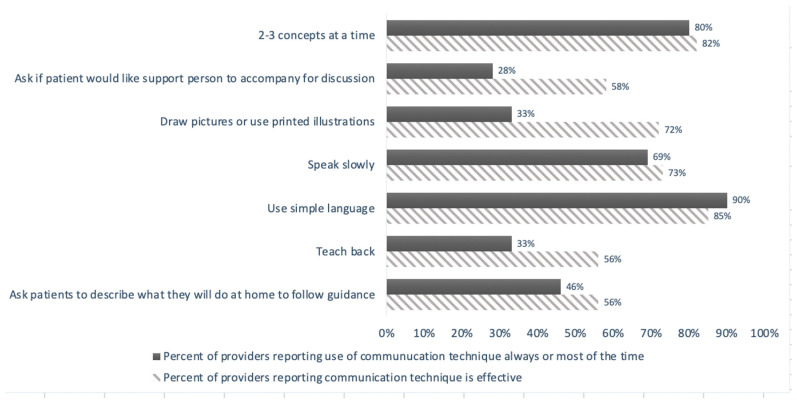
Use and effectiveness of American Medical Association’s 7 basic communication techniques in practice (*n* = 40). Assessment of provider use and perceived effectiveness of AMA’s 7 basic communication techniques; see Rozier et al., 2011, for previous assessment of these techniques in practice by oral health providers.

Looking more closely at the dental provider survey responses pertaining to just their use of and perceived effectiveness of the AMA’s seven basic patient communication techniques (Figure 2), five of these communication techniques presented are specific to interpersonal communication and the remaining two are teach-back techniques. A majority of providers indicated that they use three of the five interpersonal communication techniques all or most of the time, including using simple language (90%), presenting two–three concepts at a time (80%), and speaking slowly (69%). However, fewer reported asking patients if they would like to be accompanied by a support person for discussion (28%) and drawing pictures or using printed illustration (33%) all or most of the time. A minority of providers reported using either of the teach-back techniques, with only 33% reporting use of teach-back all or most of the time and only 46% reporting asking patients what they will do at home to follow guidance. Additionally, most providers reported not using some of the communication techniques, even though they believed the techniques were effective—such as drawing pictures or using printed illustrations, using teach-back, and asking patients if they would like their support person to accompany them to their appointment. 

### 3.3. Provider/Patient Communication Barriers

In the telephone interviews, many providers reported that a key barrier was that they had not received in-depth provider-patient communication and OHL training in professional school or continuing education programs. Most OHL experts noted that clinical dental education was more focused on the “hard skills” of technical procedures rather than the “soft skills” of providing effective patient-centered communication and education.

From the survey, all of the dentists, most of the dental hygienists (70%), and more than half of the dental assistants (56%) reported that they had experienced problems communicating with patients. Dental providers and OHL experts identified major barriers to effective provider-patient communication from the pre-survey and the telephone interviews, summarized as: (1) patient/caregiver-side communication barriers and (2) provider-side communication barriers (Table 3).

#### 3.3.1. Patient/Caregiver-Side Communication Barriers

The major patient/caregiver barrier to provider-patient communication that dental providers identified from the survey was that the patient/caregiver does not follow the provider’s instructions (56%), “no matter how well they were explained.” One-third to one-half of dental providers interviewed noted additional barriers underlying the patients’ inability to follow recommended oral health practices, including patients’ lack of understanding of oral health information, language and cultural barriers between providers and patients, and patients’ lack of interest in or prioritization of their oral health.

Dental providers referenced specific disadvantaged patient populations with whom they experienced more frequent challenges in provider-patient communication (Table 4). These populations included patients with limited English proficiency (65%), people with cognitive disabilities (54%), elderly (42%), people with limited education (35%), Deaf or hard of hearing (31%), and early childhood age groups (31%). Experts also noted that patients with limited English proficiency experienced the greatest oral health communication barriers due to cultural differences that often co-occur with language barriers and indicated that most dental practices do not adequately use translation/interpretation services.

#### 3.3.2. Provider-Side Communication Barriers

From the telephone interviews, many dental providers and OHL experts cited provider-side barriers to patient communication and education, particularly pertaining to limitations in provider training and clinical practice. Dental providers and OHL experts noted the lack of OHL communication training and proficiency requirements in professional schools and continuing education. They identified clinical practice constraints such as the inadequate time providers have for patient education during the patient encounter, limited reimbursement for delivering patient education, the paucity of high-quality patient education materials in needed languages and accessible formats, and logistical and financial difficulties to access translation/interpretation services. Public practice providers were more likely to underscore the need for technological educational resources such as video/DVD players and tablets, high-quality educational materials in a variety of languages, and translators/interpreters knowledgeable about oral health. One public practice provider discussed these structural challenges, saying:

“(The) most obvious barrier is language (…) Time is also the issue. Even if you can delegate, it’s a system issue, if you delegate to dental staff to do the education, it’s taking up chair time/office space (…) Also, many providers have limited training on health communication techniques.”

(Dentist, Public Practice)

Private practice providers were more likely to cite the lack of time and resources for patient education and reimbursement structures that prioritize dental procedures and treatment over patient education; this was described by one private practice dental assistant:

“I would like to have more time and more patient cooperation to learn. (We also) need more (patient education) resources that explain oral health and dental procedures in simple terms and that have a positive tone.”

(Dental Assistant, Private Practice)

Some of the final provider interviews overlapped with the beginning of the COVID-19 pandemic in California. Although questions about the pandemic’s impact were not included in the study instruments, we note that providers interviewed during February and March 2020 commented that practice disruptions greatly exacerbated communication problems with patients and that guidance was urgently needed to address these new patient communication barriers, both in the changed practice setting and remotely, including tele-dentistry strategies.

### 3.4. Communication Facilitators for Dental Providers

Dental providers interviewed suggested key interventions that could improve provider-patient communication by addressing the barriers they identified. They highlighted the need for more training on OHL communication techniques in professional schools and continuing education. They cited the need for patient-centered clinical practice supports, including more provider time and reimbursement incentives for delivering patient education, translator/interpreter services, high-quality low-cost educational materials in accessible plain-language formats (including videos and print materials) and in a variety of languages. In addition, they wanted information about ways to ensure a patient-friendly office. Dental providers highlighted the foremost importance of the concept of cultural humility: developing trusting, high quality, and professional relationships with patients, understanding the patient’s emotional motivators, and empowering patients to engage in preventive health behaviors. Many providers expressed their hopes that better OHL training, high-quality educational materials, and clinical supports could improve their patient communication and facilitate patients’ trust in providers, and their understanding and motivation to achieve recommended prevention and treatment goals, as described by one dentist:

“My dream would be to have California take the lead on a more innovative Medi-Cal (Medicaid—a healthcare program for people with low-incomes) Dental, create more (financial) incentives for positive oral health outcomes. Right now, it is geared to treating disease and procedures. Need to flip to outcomes based—use (performance-based) incentives for patients and providers to become healthier—achieve scalable results.”

(Dentist, Public Practice)

OHL experts recommended further structural changes in dental professional training and clinical practice and beyond. They included incorporating OHL curricula in dental professional schools and continuing education through dental professional societies, adding OHL competency requirements for clinical license examinations and/or renewal, and expanding OHL training to medical providers and health coaches. The range of suggestions for training was encapsulated by one OHL expert:

“We need to make top-down changes, starting the changes with accrediting bodies for medical/dental education, (…) board exams, (…) license recertification, and clinical quality measures and financial incentives to include good health literacy and oral health literacy practices. There are some individual medical/dental schools and scattered continuing education courses that may include health literacy/oral health literacy, but that only reaches a small subset of medical/dental providers, particularly those who are already aware of the issues. To draw in new participants, it’s helpful to tag on oral health literacy to other conferences (e.g., general pediatrics) and present compelling cases to show how oral health is critical to overall health.”

(OHL expert)

Experts also discussed the need for training the entire dental team in effective OHL strategies, including establishing a patient-friendly environment, using OHL communication techniques, and using oral health-specific translator/interpreter services such as via phone/video services and natural language processing software. Dental providers in public practice and OHL experts also recommended expanded community oral health education, through integrative dental and medical care models particularly during early childhood (e.g., in pediatrics), community events and schools from early childhood through high school, to raise the public’s knowledge of oral health and help patients feel more comfortable with dental care.

## 4. Discussion

The global epidemic of poor oral health and increasing oral health inequities presents major public health challenges. Despite substantial evidence that most oral health problems are preventable, national and global oral health goals have not been achieved. Two decades of research has identified OHL as a major determinant of oral health status through multiple interrelated factors that include people’s understanding about oral health, providers’ knowledge of current scientific evidence, the quality of dental providers’ communication with patients, and the “patient-friendliness” of dental practice environments. Although early OHL research focused primarily on individuals’ abilities to understand oral health information, during the past decade, an increasing number of studies have investigated the impact of dental providers’ communication with patients. This reflects the perspective that oral health is not just an individual responsibility but an issue of social justice and health equity impacted by a myriad of factors across social-ecological levels [4]. These studies have been conducted primarily in the US and have shown that although dental providers use some recommended communication techniques, such as speaking slowly, using simple language, and visual models, overall, dental providers reported using less than half of recommended communication techniques with their patients, including the important teach-back approach [10,21,25,26,27,28]. Research in the UK has also demonstrated that providers generally provide education as “ad hoc” lectures, use few visual aids, and rarely provide take-home educational materials [38].

Results from our study showed that providers greatly valued their communication with patients and believed they were effective communicators. During the interviews, we were struck by the care and concern providers expressed for their patients and their strong desire to help them improve their oral health. We were also struck by the frustration they expressed about communicating effectively with patients: the vast majority cited multiple barriers. A notable finding was that providers most often attributed communication problems to patient-side, rather than provider-side barriers. Table 3 shows that one-third or more of dental providers cited six out of the seven patient-side barriers, but only one out of the five provider-side barriers. In other words, providers focused on patient issues such as “not understanding” oral health information or “not being interested” in their oral health.

This perception aligns with the historic “patient deficit” health literacy approach and signals the critical need for provider OHL training about their key role to help patients understand and improve their oral health. In this study, providers tended to believe their communication practices were quite good, but despite that, the patient or caregiver does not follow their instructions. Yet, few providers indicated they used teach-back—the communication technique most likely to get positive results. This suggests that providers may not recognize their need for training to use recommended communication techniques and how those techniques could significantly help them and their patients. In the larger scientific context, the concept of health literacy has undergone a transformation from placing the communication burden on the patient to transferring that responsibility to the provider [12,39].

Study findings described providers’ knowledge and use of OHL techniques. Most providers were familiar with the term “oral health literacy,” but few fully understood that it goes beyond patients’ comprehension to include the quality of provider communication with patients and the patient-friendliness of dental practices. Because OHL is still a new concept to most providers, we believed it was important to follow up on online survey responses with individual interviews. For example, although two-thirds of providers reported in the pre-survey that they had taken a patient communication course in professional training, probing during the interviews revealed that such training did not generally go into depth about OHL and its recommended communication techniques. Methodologically, this study highlights the importance of using in-depth interviews rather than just relying on provider survey responses.

In this study, providers’ reported OHL practices were similar to findings in prior OHL research. California providers reported using 6 of AMA’s 18 recommended communication techniques and 3 of AMA’s 7 basic communication techniques. Of the 18 communication techniques, providers most often reported using simple language, speaking slowly, presenting only two–three topics at a time, and using visual models. There was less routine use of other recommended techniques, such as teach-back, printing out instructions, referring patients to the Internet or other resources, using videos or DVDs, or following up with patients after a dental visit, etc. Of the seven basic communication techniques, providers indicated that they most often used three of the basic techniques categorized as interpersonal communication, including using simple language, presenting two–three concepts at a time, and speaking slowly. There was less routine use of other recommended basic techniques, such as asking patients if they would like to be accompanied by a support person for discussion, drawing pictures or using printed illustration, and teach-back strategies, including asking patients what they will do at home to follow guidance. Of the five assessment and educational strategies we asked about, a majority of providers interviewed indicated they were knowledgeable of and used one of the five strategies (CAMBRA), but were unaware of or did not use Family Oral Health Education, anticipatory guidance, teach-back, or motivational interviewing. For less-frequently used techniques, providers also reported “less confidence” in the effectiveness of those techniques, indicating a gap in understanding about OHL and its associated communication techniques. Although half of providers reported that they had assessed their practice for patient-friendliness, they did not confirm how they did so, suggesting that the assessment may have been intuitive, rather than based on recommended processes, such as those in the “Health Literacy Environment 2” tool [20]. For example, providers could assess whether their staff provides patients with plain language information and translation services or helps filling out forms. In this study, providers wanted information about these processes.

The study’s results also corroborate prior studies [21,25,26,27,28] and expert recommendations pertaining to key communication challenges facing providers. Provider challenges included the lack of professional training in OHL communication, lack of accessible, in-language patient educational materials, lack of translator/interpreter services, and lack of provider time and financial incentives for patient education. Lack of OHL can lead to poor oral hygiene at home and uninformed decisions about fluoridated tap water and fluoridated toothpaste, and inappropriate amounts of toothpaste used. A 2020 study by Avenetti et al. comments on the lack of knowledge that parents/caregivers have regarding fluoride use, fluorosis, and best oral health behaviors for their children. For example, parents purchasing toothpaste were often confused by marketing strategies and labels on non-fluoridated toothpaste such as “safe for babies” or on fluoridated toothpaste encouraging parents to consult a dentist or physician for use in children under 2 [35]. For young children in particular, promoting OHL is vital because the prevention of caries is primarily in the hands of the parents/caregivers. Furthermore, their efforts regarding the oral health of their child serves to establish oral health behaviors that will impact the child’s life course [5,35].

Providers and national experts in this study recommended that achieving marked improvements in patient communication requires prioritizing more OHL professional training (academic and continuing education) in basic communication techniques and access to low-cost, high-quality plain language and in-language educational materials and tools. They also recommended better access to translator/interpreter services knowledgeable in oral health, and greater financial incentives to incentivize providers to provide patient education.

Additionally, providers and national experts recommended further systematic changes, including curricular requirements that OHL communication be taught in dental professional schools, that content be included in continuing education, and that there be assessment of provider performance of OHL competencies. This might include strategies in professional continuing education, as peer or expert performance evaluations of mock patient interviews, or adding content to state licensing exams. They also mentioned that wider access to dental insurance is needed to extend oral health education to more people, especially to disadvantaged groups such as people with limited English proficiency, people with cognitive disabilities, older adults, people with low literacy, people who are Deaf or hard of hearing, and children. Providers and experts also emphasized the importance of promoting inter-professional collaboration about oral health literacy. Not only do dentists need to be educated on how to teach and support patients, but other medical professionals need training as well [35,40].

Having additional patient educators, such as community health workers or promotoras may benefit dental practice communication [35,41]. In many practices, the pressure on dentists and hygienists to see a high volume of patients for reimbursable treatments may not allow them adequate time to provide “soft” communication and literacy support. Community health workers/promotoras can help share the workload by providing the needed culturally relevant communication and support as part of the care team.

Furthermore, both providers and national experts recommended going beyond dental practices to extend oral health education to the broader community level, including oral health patient education in medical practices, especially in pediatric and family medicine practices [40,42], mass media communication, and oral health education in schools from early childhood through high school. This study adds to the limited global literature about OHL and dental provider communication and is the first to examine California’s dental provider/national OHL expert perspectives on oral health communication.

The study has limitations. Due to its convenience, purposive sample, study results for knowledge and use of educational and communication techniques and other findings are not widely generalizable. However, it includes 44 in-depth interviews that achieved thematic data saturation and 40 completed pre-surveys with the same providers, across the major groups of dental professionals. This is complemented by six interviews with OHL experts, which together with provider interviews, present a wide range of perspectives from California dental providers and OHL national subject matter experts.

In this study, we included pre-surveys to gather background information about provider and expert characteristics and perspectives prior to conducting the in-depth interviews. We have found this a useful technique to reduce the time needed in the qualitative interview, and to provide background information to the interviewer about interviewees’ perspectives to be explored more deeply in the qualitative interview. This approach can also help identify possible weaknesses of relying on survey responses without further probing them in interviews. For example, although most providers reported in the pre-survey that they were familiar with the term “health literacy,” during the interviews, it was apparent that they had a very limited view of that concept—limiting it to just the literacy skills of patients. We recommend the use of “pre-surveys” prior to conducting key informant interviews.

The study questions were informed by those in national and state studies of OHL among dental professionals (content validity), with the addition of California-specific questions including those covering suggestions for training, patient resources, and payment. In addition, responses from KIs in each dental professional subsample achieved “data saturation” on major issues and results were aligned with those found in other larger, quantitative studies. This study addressed perspectives of dental providers. More research is needed about patient and general public perceptions of the dental providers’ communication skills. Other variables related to patient-provider communication could be explored, such as the “Oral health-related quality of life (OHRQoL)” tool, an assessment intended to help patients and providers make better shared decisions about treatments [43].

Smaller categories of techniques help reduce the overlap in some of the AMA’s 18 communication techniques, put more focus on priority techniques, and make it easier to incorporate techniques into more practical educational interventions, including those using digital channels, such as through networks of dental providers.

This study was intended to convert research into practice and provided rich, detailed recommendations about preferences for training and supportive resources. The CDPH OOH is now implementing these results and those of past studies in a collaborative effort with oral health stakeholders to create OHL continuing education trainings beginning in 2021, and a toolkit of OHL resources for dental providers and community organizations [29]. The American Dental Association’s National Advisory Committee on Health Literacy in Dentistry is considering ways to adapt the proposed trainings and toolkit resources to reach national dental provider and community audiences. Such interventions could inform similar efforts in other countries.

The COVID-19 pandemic has greatly increased provider-patient communication barriers and widened oral health disparities, underscoring the urgent need to draw on the substantial OHL research and practice guidance of the past two decades to train providers and accelerate the search for innovative, effective patient communication interventions.

## 5. Conclusions

Two decades of OHL research suggest that gaps in people’s understanding of oral health and providers’ low use of recommended communication techniques with patients are important factors contributing to poor oral health in the US and globally. Fortunately, such research also provides evidence-based recommendations for oral health interventions. This study focused on dental providers’ reported communication practices and perceived barriers and facilitators to improved communication. Results showed major gaps between current dental provider communication practices and those recommended in prior research and by medical and dental professional organizations—similar to the gaps found in other studies. The in-depth interviews in this study add new knowledge about providers’ focus on patient OHL limitations vs. their own patient communication limitations and the many barriers providers experience in improving their communication skills and incorporating them into a busy practice. The study results detail specific provider and expert recommendations related to OHL training and resources to improve OHL communication. These results underscore the larger issue that the epidemic of oral health problems will require actions at all levels of society from education of individuals to policy changes. This study was intended to focus on the dental provider component of the oral health ecosystem with the intent to use the findings to create interventions to improve dental provider OHL and patient communication, and ultimately to adapt interventions to larger numbers of dental providers, to medical oral health providers, to community organizations, and to advance policy changes. In our view, using research to identify issues, while simultaneously generating solutions relevant to specific contexts—in this case, dental providers in the US state of California—is the most powerful way to address complex problems, such as oral health.

## Figures and Tables

**Table 1:** Provider demographics and communication background (*n* = 40).

Descriptive Characteristic	All Providers
	*n* (%)
Gender	
Male	12 (30%)
Female	28 (70%)
Race/Ethnicity *	
White	24 (60%)
Asian/Pacific Islander	11 (25%)
Hispanic/Latino	4 (10%)
Black/African American	1 (3%)
Other	2 (5%)
Years in the field	
Average years in field (range)	22 (1.5–49)
Current practice type *	
Private practice	25 (63%)
Public practice/FQHC	15 (37%)
Rating of HL and communication training	
Very good	13 (33%)
Good	21 (53%)
Poor	4 (10%)
Very poor	1 (3%)
Not sure	1 (3%)
Ever taken a communication course outside of professional training	
Yes	26 (65%)
No	14 (35%)
Interested in attending CE on communication strategies	
Yes	28 (70%)
Maybe	10 (25%)
No	2 (5%)
Ever assessed practice for patient friendliness	
Yes	20 (50%)
No	15 (38%)
Unknown	5 (13%)

* Non-exclusive responses, with participants allowed to select more than one response option.

**Table 2:** Provider knowledge and use of five types of oral health assessment and educational strategies in practice * (*n* = 44).

Communication Technique	Knew of It and Used It in Practice (%)	Knew of It and Did Not Use It (%)	Did Not Know of It (%)
Caries risk assessment (CAMBRA)	26 (59%)	12 (27%)	6 (14%)
Anticipatory guidance	19 (43%)	2 (5%)	23 (52%)
Family oral health education (FOHE)	13 (30%)	2 (5%)	29 (66%)
Motivational interviewing	15 (34%)	6 (14%)	23 (52%)
Teach-back	14 (32%)	6 (14%)	24 (55%)

* Results based upon provider self-report of knowledge and use of five types of oral health assessment and educational strategies during qualitative interviews. Incorrect descriptions of the technique were categorized as “did not know it”.

**Table 3:** Communication challenges for providers: patient/caregiver-side challenges and provider-side challenges (*n* = 40).

Communication Measure	All Providers *n* (%)
Greatest challenges in communicating with patients/caregivers (P/C) *	
Patient/caregiver-side challenges	
P/C does not follow instructions, regardless of how well explained	22 (56%)
Cultural beliefs are a barrier to patient/caregiver understanding	20 (51%)
Language is a barrier to patient/caregiver understanding	16 (41%)
P/C is not that interested in OH education	15 (38%)
P/C does not understand OH information	14 (36%)
P/C is afraid of the dentist/oral health provider	13 (33%)
P/C has trouble communicating due to treatment in mouth/oral cavity	5 (13%)
Provider-side challenges	
Other staff, no background in communication techniques	14 (36%)
Not enough time	10 (26%)
Not enough background in communication techniques	4 (10%)
Does not have simple educational resources to give patients/caregivers	4 (10%)
Other	9 (23%)

* P/C: Patient/caregiver.

**Table 4:** Communication help needed for providers by type of patient/caregiver (*n* = 40).

Communication Measure	Providers Reporting That They Need Help Communicating *n*(%)
Need help communicating with patients	
Yes	27 (69%)
No	12 (31%)
Patient groups providers need help communicating with:	
Persons with limited English proficiency	17 (65%)
Persons with cognitive disabilities	14 (54%)
Elderly patients	11 (42%)
Those with low levels of education	9 (35%)
Deaf or hard of hearing individuals	8 (31%)
Children under age 5	8 (31%)
Other	6 (23%)

## Data Availability

The data presented in this study are available on request from the corresponding author. The data are not publicly available for the audio recorded interviews due to confidentiality. However, de-identified transcripts can be provided upon request.
